# Mathematical Modeling of Protein Misfolding Mechanisms in Neurological Diseases: A Historical Overview

**DOI:** 10.3389/fneur.2018.00037

**Published:** 2018-02-02

**Authors:** Felix Carbonell, Yasser Iturria-Medina, Alan C. Evans

**Affiliations:** ^1^Biospective Inc., Montreal, QC, Canada; ^2^Department of Neurology & Neurosurgery, McConnell Brain Imaging Centre, Montreal Neurological Institute, Montreal, QC, Canada; ^3^Ludmer Centre for NeuroInformatics and Mental Health, Montreal, QC, Canada

**Keywords:** misfolded protein, prion-like hypothesis, mathematical modeling, neurodegeneration, therapeutic interventions

## Abstract

Protein misfolding refers to a process where proteins become structurally abnormal and lose their specific 3-dimensional spatial configuration. The histopathological presence of misfolded protein (MP) aggregates has been associated as the primary evidence of multiple neurological diseases, including Prion diseases, Alzheimer’s disease, Parkinson’s disease, and Creutzfeldt-Jacob disease. However, the exact mechanisms of MP aggregation and propagation, as well as their impact in the long-term patient’s clinical condition are still not well understood. With this aim, a variety of mathematical models has been proposed for a better insight into the kinetic rate laws that govern the microscopic processes of protein aggregation. Complementary, another class of large-scale models rely on modern molecular imaging techniques for describing the phenomenological effects of MP propagation over the whole brain. Unfortunately, those neuroimaging-based studies do not take full advantage of the tremendous capabilities offered by the chemical kinetics modeling approach. Actually, it has been barely acknowledged that the vast majority of large-scale models have foundations on previous mathematical approaches that describe the chemical kinetics of protein replication and propagation. The purpose of the current manuscript is to present a historical review about the development of mathematical models for describing both microscopic processes that occur during the MP aggregation and large-scale events that characterize the progression of neurodegenerative MP-mediated diseases.

## Introduction

**Figure 1:**
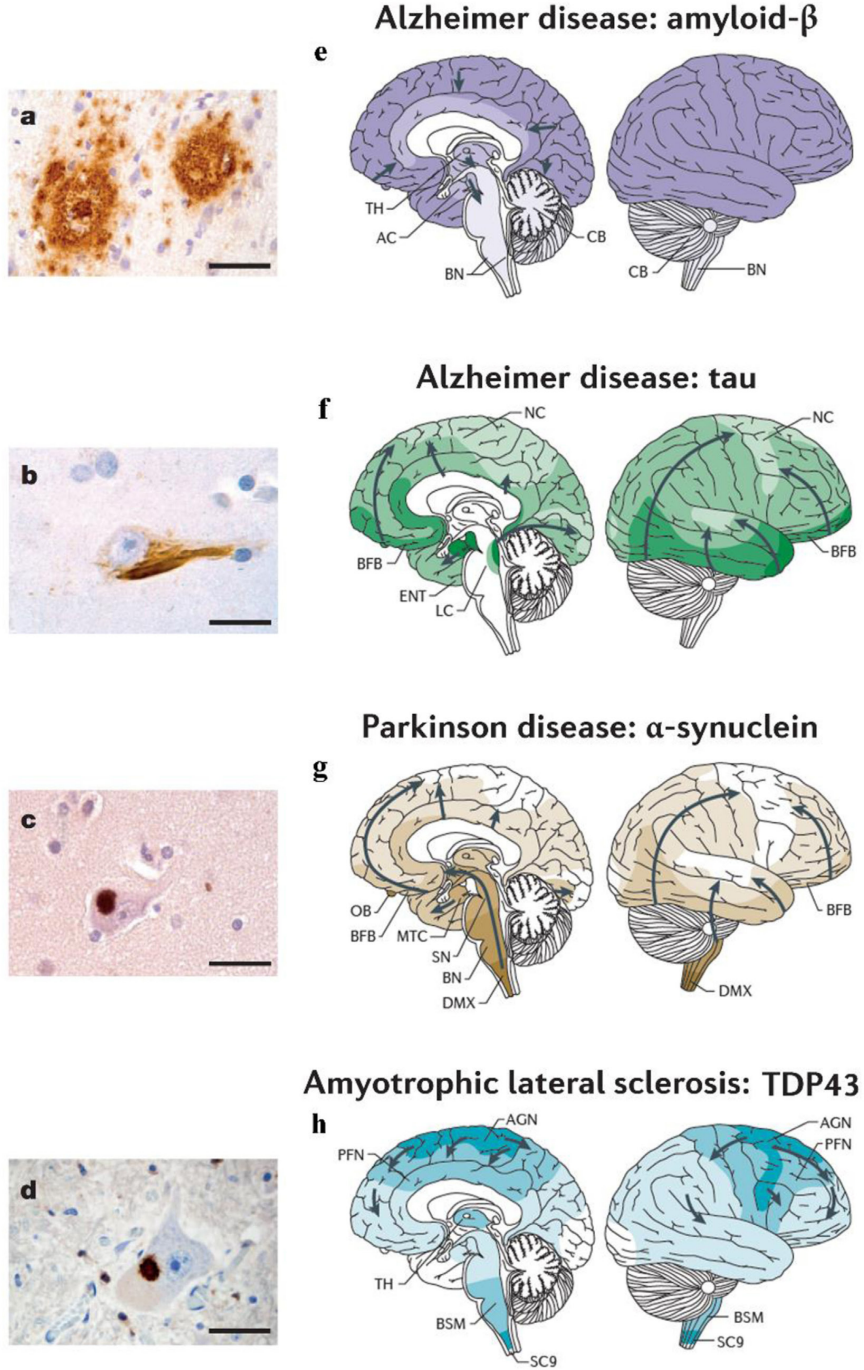
Different neurodegenerative disorders present disease-specific MPs and characteristic anatomical progression patterns. **(A)** Aβ plaques in the cortex of an Alzheimer’s disease (AD) patient. **(B)** Tau neurofibrillary tangle in a neuron of an AD patient. **(C)** α-synuclein inclusion in a neuron from a Parkinson’s disease (PD) patient. **(D)** TDP-43 inclusion in a motoneuron of the spinal cord from a patient with ALS. Scale bars are 50 mm in **(A)** and 20 mm in **(B–D)**. **(E)** In Alzheimer’s disease (AD), Aβ deposits are first observed in the neocortex (NC) and are then detected in all cortical, diencephalic and basal ganglia structures (in a caudal direction) and in the brainstem, and occasionally in the cerebellum (8, 9). **(F)** Tau aggregates develop in the locus coeruleus, then in the transentorhinal and ENT regions and subsequently in the hippocampal formation and in broad areas of the NC (10, 11). **(G)** In PD, the progression of α-synuclein pathology follows an ascending pattern from the brainstem to the telencephalon (9, 11). The earliest lesions can be detected in the olfactory bulb, and in the dorsal motor nucleus of the vagus nerve (DMX) in the medulla oblongata. At later stages, the α-synuclein aggregates are found more rostrally through the brainstem *via* the pons and midbrain, in the basal forebrain and, ultimately, in the NC. **(H)** In ALS, initial TDP43 inclusions are seen in the agranular motor cortex (AGN), in the brainstem motor nuclei of cranial nerves XII–X, VII and V, and in α-motor neurons in the spinal cord. Later stages of disease are characterized by the presence of TDP43 pathology in the prefrontal neocortex (PFN), brainstem reticular formation, precerebellar nuclei, pontine gray, and the red nucleus. Subsequently, prefrontal and postcentral neocortices, as well as striatal neurons, are affected by pathological TDP43, before the pathology is found in anteromedial portions of the temporal lobe, including the hippocampus (9, 12). AC, allocortex; BFB, basal forebrain; BN, brainstem nuclei; BSM, brainstem somatomotor nuclei; ENT, entorhinal cortex; MTC, mesiotemporal cortex; SC9, spinal cord gray-matter lamina IX; SN, substantia nigra; TH, thalamus. Figures **(A–D)** and **(E–H)** were adapted with permission from Ref. (13, 14), respectively.

Proteins, large molecules composed by amino acids, play a central role in biological processes and constitute the basis of all the living organisms. During different conformational phases of the proteins, their folding into compact three-dimensional structures is a remarkable example of biological self-assembly and complexity (1). Only correctly folded proteins have long-term stability in crowded biological environments, while a folding failure is traditionally associated with a variety of pathological conditions (1). Proteins that fail to configure properly are called misfolded proteins (MP). In particular, these are thought to disrupt the function of cells, tissues, and organs (2, 3) and have been causally related to multiple conformational disorders, such as Prion diseases, Alzheimer’s disease (AD), Parkinson’s disease (PD), Creutzfeldt–Jakob disease, amyotrophic lateral sclerosis (ALS), and several other human degenerative disorders (see Figure 1) (1, 4–7).

Despite the biological importance of its negative effects, the mechanisms underlying MP seeding, aggregation, propagation, and/or effective toxicity spreading are not totally understood (15–17) and have been the subject of scientific controversy for decades (14, 18, 19). The high complexity of the underlying processes, as well as the difficulties to extrapolate their effects from microscopic (e.g., molecular) to macroscopic (e.g., organs) scales, have become central obstacles toward the identification of conformational disease-specific triggering events. As a consequence, discrete advances have occurred in the development of effective therapeutic interventions.

For several decades, we have seen the emergence of different mathematical approaches aimed to complement our understanding of the biological mechanisms that lead to MP-related diseases. In a broad manner, mathematical models can be methodologically categorized into two different classes. Namely, a large class of models designed to reproduce molecular-level processes (e.g. seeding, aggregation, short-range spatial spreading in any biological tissue), and a relatively small class of models that account for inter-regional macroscopic interactions (e.g., long-range MP propagation in the human brain). The former class of models have been traditionally developed within the field of chemical kinetics, while the latter one is almost restrictive to neuroimaging studies. Unfortunately, both research fields tend to follow divergent paths. It is not hard to note that neuroimaging-based models invariably follow a single (large-scale) perspective, barely referring to pertinent models coming from the chemical kinetics field.

Motivated by the current lack of an integrative methodological perspective, in this article, we provide a comprehensive historical overview of mathematical models aimed to characterize MP seeding, aggregation, and propagation processes. The manuscript is organized in three sections. The first section offers an overview of models describing prion dynamics, particularly seeding, aggregation, and short-range spatial spreading processes. The second section reviews recent advances on the modeling of prion-like dynamics associated with neurological disorders. The third section highlights important aspects on modeling strategies for the development of drugs and therapeutic interventions in neurological diseases.

## Prion Dynamics

### Prion Aggregation: One-Dimensional Models

**Figure 2:**
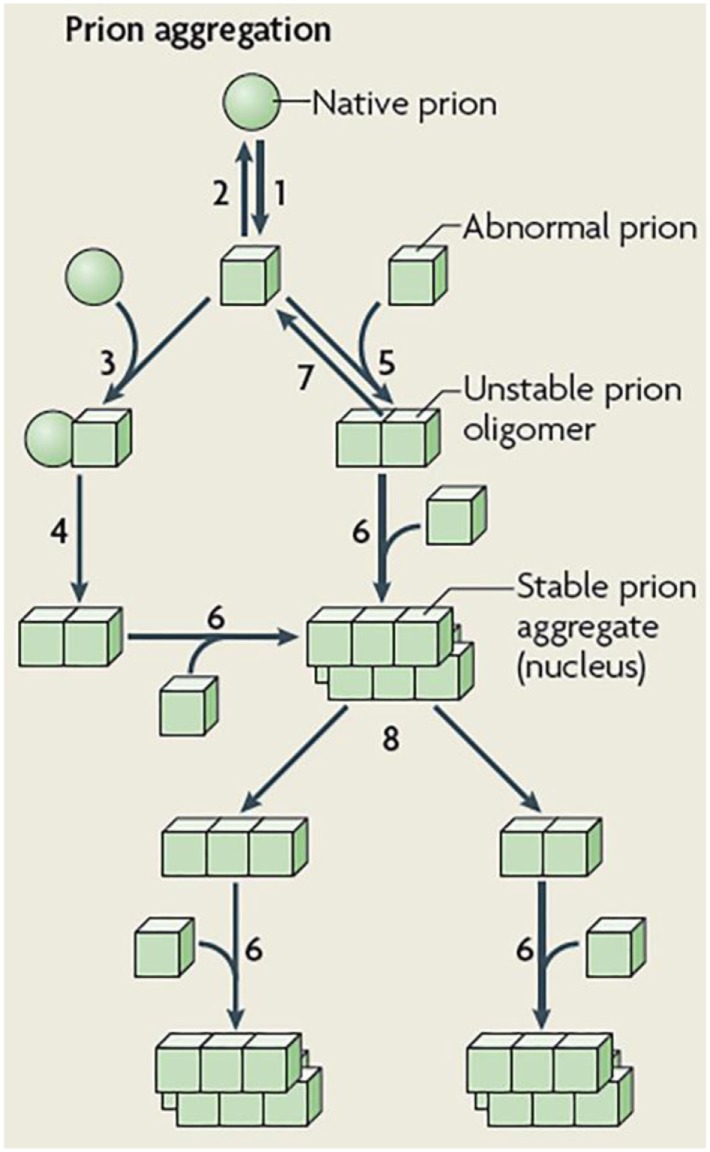
Schematic representation of prion aggregation mechanisms. Native (sphere) prion molecules undergo conformational changes that lead to an abnormal (cube) configuration (Step 1). This event is unfavorable because the abnormal configuration is either unstable (Step 2) or sensitive to clearance. According to the template-assisted model (24), prions in their abnormal configuration interact with native prions (Step 3) and convert them into the abnormal configuration (Step 4). The NPM proposes that abnormal prions can interact with molecules in a similar state (Step 5), the oligomeric species formed are unstable and grow by the incorporation of abnormal prion molecules (Step 6) and dissociate (Step 7) until a stable nucleus is formed. Such a stable prion aggregate can then grow indefinitely from one or both ends and can also break into smaller fragments (Step 8) that act as new nuclei. Figure reproduced from Brundin et al. (25) with the permission of the journal.

Pioneering mathematical models on prion dynamics (20) mainly focused on simulating the biological mechanisms of prion replication/aggregation previously described in Ref. (21, 22). By using ordinary differential equations (ODEs), Eigen (20) proposed a prototype model for the autocatalysis mechanism of Prusiner (21, 23), which intended to explain the conversion of a typical prion protein (denoted by PrP^C^) into an infectious agent protein (usually denoted by PrP^Sc^). The autocatalysis mechanism, usually called either the template-assisted model or the heterodimer model (24) (see steps 3 and 4 in Figure 2), features a low rate production of PrP^Sc^ by spontaneous conversion of PrP^C^. The PrP^Sc^ protein then catalyzes the conformational change of PrP^C^ by forming a heterodimer, which in turn, dissociates at faster rate into two molecules of PrP^Sc^. However, the steady-state analysis (20) of the system describing this mechanism revealed unrealistic requirements for the rate constants. Consequently, the heterodimer model would be, for instance, unable to replicate the long incubation period typically found in prion diseases.

In the same study, Eigen (20) extended the heterodimer model to a framework of a cooperative catalysis mechanism. In this new scenario, a threshold effect on the concentration of PrP^Sc^ would determine whether the system maintain the healthy PrP^C^ state (i.e. stable concentration of PrP^C^) or turn (by an autocatalysis mechanism) into a state of exponential production of PrP^Sc^. Despite that extended model allows for a more meaningful values range for the rate constants, it is still unable to simulate realistic scenarios of prion replication. In order to overcome this limitation, Eigen (20) showed the need to suppress the linear autocatalysis component from the model. In fact, another disadvantage of the cooperative catalysis model is its tendency to generate dynamics of globular aggregates of PrP^Sc^ (26). This behavior clearly contrasts with the observations of fibrils of pathogenic PrP^C^ actually conforming geometrically linear on a macroscopic level (22). Nevertheless, with the aim of keeping the argument of a threshold effect, Eigen (20) also simulated the Lansbury’s mechanism (22) of plaque formation. In this mechanism, prion replication does not explicitly require a catalysis process but relies on nucleated polymerization. In this way, the steady-state analyzes in Ref. (20), in conjunction with the *in vitro* models developed by Harper and Landsbury in Ref. (27), yield a mathematical formalization of the prion replication mechanism that is currently known as the nucleated polymerization model (NPM) (26, 28).

In summary, the NPM is characterized by four key aspects: (i) no replication occurs below a critical threshold of protein concentration (i.e., polymerization is very slow below a critical size); (ii) a lag time before polymerization for protein concentrations just above the critical threshold; (iii) a relatively rapid polymerization for protein concentrations well above the critical size; and (iv) the slow nucleation process can be bypassed by the introduction of exogenous nucleus or seed (27, 29). A formalization of a mathematical model describing the dynamics of the NPM is due to Nowak et al. (28). In particular, that model describes how PrP^Sc^ aggregates can either elongate by the incorporation of a PrP^C^ monomer or break into two new aggregates. These mechanisms of elongation and fragmentation of PrP^Sc^ are typically expressed by an infinite set of ODEs (see Eq. A1 in Appendix in Supplementary Material). As noted by Nowak et al. (28) and Masel et al. (26), that system of infinite ODEs can be closed by summation to yield an ODEs system of only three equations (Eq. A2 in Appendix in Supplementary Material). Remarkably, Nowak et al. (28) realized, for the very first time, the analogy between this model and popular epidemiological models for describing virus dynamics (30).

A detailed analysis [see Ref. (28)] of the Eq. A1 revealed a mechanism whereby aggregation could be initiated from a PrP^Sc^ monomer at a uniform rate, thus neglecting the (highly probable) possibility of aggregation from a nucleated seed. Based on this observation, Nowak et al. (28) introduced a modified model that allows for a more realistic assumption: all PrP^Sc^ aggregates below a critical threshold size would become unstable and quickly dissociate into pieces that return to a PrP^C^ state. Therefore, a realistic aggregation mechanism would require a nucleation-based seed of at least a critical threshold size. Unfortunately, that modified model [see Appendix B in Supplementary Material in Ref. (28)] was not closed by summation as in the case of Eq. A1 and required further approximations. Nevertheless, Masel et al. (26) extended Nowak’s NPM for covering the possibility of a nucleated seed [see Eq. 8 in Ref. (26)] while also reducing the number of kinetic rates and making the system closed by summation. As in the case of Eq. A2, the Masel’s NPM can be also described by an epidemiological-like system of three ODEs (see Eq. A3 in Appendix in Supplementary Material). In comparison with the original formulation of Nowak et al. (28), the Masel’s model not only provided important mathematical simplifications but also facilitated the validation of the NPM dynamics with *in vitro* data.

Several modifications and extensions to the NPM has been proposed during the last two decades (31–36). A remarkable one was given by Greer et al. (33), which, rather than modeling the PrP^Sc^ dynamics through an infinite set of differential equations, considered a continuum of possible fibril lengths, written down by a transport partial differential equation (PDE). The PDE formalism turned out to be more accessible from the mathematical point of view and yielded three-dimensional epidemiological-like models. In addition, the PDE formalism enables the study of different aspects of prion dynamics, such as the distribution of prion fibrils and fragmentation processes (33). By relying on the Greer’s approach, Prüss et al. (34) provided a detailed characterization of the epidemiological-like behavior of the NMP. By making an analogy with the basic reproductive ratio (30) used in epidemiological theory, Prüss et al. (34) defined a constant *R_0_* as the number of secondary infections produced on average by one infectious prion (i.e., PrP^Sc^). Thus, if *R_0_* ≤ 1, the prion replication stops and the disease-fee equilibrium state turns out globally asymptotically stable. On the contrary, if *R_0_* > 1, the prion replication persists as an asymptotically stable disease state.

As in the case of the discrete formulation of the NPM, some studies (37, 38) have focused on extending the Greer’s continuous fibril length modeling (33) to more general frameworks (e.g., size-dependent kinetic parameters). Despite the mentioned advantages of the continuous formulation, some efforts have been put on investigating its mathematical connection with the discrete model, as well as the biological implications of such generalization (39). A more recent study (40) has demonstrated that the discrete formulation of the NPM can still be exploited in order to gain further insights about the dynamics of prion replication/aggregation.

### Prion Propagation: Spatial Spreading

The main motivation for considering neuronal transport of prion proteins seems to be given by early observations in animal models (41) about the latency between the appearance of PrP^Sc^ in the peripherical nervous system and its appearance in the central nervous system. This latency cannot be explained by the rate at which PrP^C^ is converted into PrP^Sc^ at specific spatial location, but it is more likely related to the rate of spread between neighboring localities (42). The first attempt to simultaneously model processes of prion aggregation and neuronal transport inside the brain was given by Payne and Krakauer (42). Indeed, Payne and Krakauer (42) extended the template-assisted model (20, 23) of prion replication by adding a spatial spreading component consisting on the statistical assemblage of PrP^Sc^ molecules with a classical diffusion process. Note that, with the introduction of this spatial spreading component, Payne and Krakauer (42) overcame the main limitation (i.e. no disease latency) of the template-assisted model. However, due to the acceptance of nucleated polymerization as a plausible mechanism for prion aggregation, incorporation of spatial spreading into the NPM seems to be a promising choice. With this aim, Matthäus (43) used the diffusion approach within the discrete NPM in order to model prion spatial spreading over simple (i.e., one-dimensional) domains like the spine or the mouse visual system (see Eq. A4 in Appendix in Supplementary Material). However, classical diffusion is better suited for modeling free movement of particles in homogenous medium, while the whole brain is a highly heterogeneous one. By acknowledging this limitation, Matthäus (43) proved that the solutions of the NPM model with one-dimensional diffusion follow a traveling wave behavior. Despite such characterization provided a clearer interpretation about the prion propagation speed along pre-defined spatial domains, the proposed model cannot be reduced to a simpler epidemiological-like system. Besides, as pointed out by Matthäus in Ref. (43), it is not realistic to extend the isotropic diffusion approach to large multi-dimensional domains. Indeed, modeling diffusion in a homogeneous medium would force prions to spread with equal speed in all spatial directions. This is unlikely to happen in practice since *in vitro* models have proved that prions spread along the neuronal pathways (44), where infection may reach distant cells at the same time or even faster than neighboring cells (43).

By using a simplified approach, Stumpf and Krakauer (45) modified the epidemiological-like configuration of the NPM in order to incorporate spatial “connectivity” features into the temporal evolution of prion kinetics. Specifically, Stumpf and Krakauer (45) used a lattice domain to model the influence of cell connectivity and cell density in several prion diseases (see Eq. A5 in Appendix in Supplementary Material). The main assumption in Stumpf–Krakauer’s model (45) is that PrP^Sc^ components spatially spreads along axons and dendrites by slow axonal transport, where the rate of spread from cell to cell depend on the connectivity strengths. To our knowledge, Stumpf and Krakauer model constitutes the first successful attempt of incorporating brain connectivity features into prion diseases models described by epidemiological-like equations.

Summarizing, Matthäus (43) and Stumpf and Krakauer (45) were the first studies that attempted to fill the gap between two different modeling scales: reaction kinetics at molecular level and spatial spreading along a large (e.g., whole brain) and complex domain. Unfortunately, these pioneering studies have received little acknowledgment in subsequent studies about proteins propagation over large-scale brain networks. Due to its close relationship with current large-scale protein propagation models, we will provide more details about the network approach over the following sections.

## Prion-Like Dynamics in Neurological Diseases

Similarly to prion diseases, several neurodegenerative diseases (e.g., AD, PD, and FTD) are pathologically associated with the presence of MP (e.g., tau, Aβ, α-synuclein; see Figure 1) (15, 17, 25, 46, 47). By using *in vitro* models, it has been demonstrated (48–50) that fibril aggregates of α-synuclein, tau and Aβ proteins self-propagate under biochemical mechanisms analogous to those described for prion aggregation/propagation. These observations in conjunction with several *in vivo* animal models (50, 51) established the founding of the so-called prion-like hypothesis of neurodegenerative progression. Under the prion-like hypothesis, the MP “infectivity” propagates from initial seed regions with a relative high concentration of pathogenic proteins to other “non-infected” brain regions. It should not be surprising that the development of mathematical models for aggregation/propagation of Aβ have followed concurrent paths with those of prion evolution.

### Nucleated Polymerization of Aβ

As in the case of prions, the NPM has been accepted as a plausible preliminary mechanism for Aβ aggregation/propagation (22, 27). However, early *in vitro* studies (27, 52, 53) suggested that the actual mechanism of Aβ aggregation might be more complex than the classical NPM. Indeed, Aβ aggregation is a mechanism likely involving the formation of intermediates soluble micelles [also called protofibrils in Ref. (53)], which are in rapid equilibrium with APP monomers. Such interaction yields domains of high local protein concentration that facilitate the formation of fibril nucleus (27, 52, 54–56). As pointed out in Ref. (54), the formation of intermediates micelles had not been previously detected because the methods for quantification of Aβ aggregation at that time (e.g. Turbidity, Thioflavin T fluorescence) were only able to detect large polymeric structures such as Aβ aggregated fibrils. Using a Quasielastic Light Scattering technique, Lomakin et al. (52) proposed an *in vitro* model that facilitates a quantitative monitoring of the kinetics of Aβ fibrils formation, and consequently, the detection of smaller polymeric structures. Based on that study, Lomakin et al. (54) formalized a mathematical model for describing the simultaneous temporal evolution of APP monomers, Aβ micelles and Aβ fibrils. While in the classical NPM the long latency phase was interpreted as the time required for nucleation, the findings of Ref. (52, 54) suggest that it is rather the time required for formation of larger Aβ protofibrils (55–57). Based on this re-interpretation of the NPM, fibrillization of Aβ would be still a nucleation-dependent process that occur under two concurrent nucleation pathways: exogenous seeds and intermediates Aβ micelles.

As in the case of NPM, Lomakin’s model involved an infinite set of ODEs, one per each fibril length. That system can be closed by summation to yield a set of four differential-algebraic equations. There, two ODEs correspond to the temporal evolution of the total length of Aβ fibrils and the total concentration of Aβ aggregates, while two algebraic equations relate (by a conservation of mass condition) the number of APP monomers and Aβ micelles with the Aβ fibrils. Although Lomakin’s model was able to replicate the temporal evolution of the mass concentration of fibrils and the fibrils length, it did not consider any fragmentation process. Despite this limitation, Lomakin’s model constituted a successful attempt to mathematically model detailed mechanisms of Aβ fibrils and intermediates assemblies of different sizes.

Realizing that experiments in Ref. (52) had been conducted under nonrealistic physiological conditions, Murphy and Pallitto (58) carried out a thoughtful *in vitro* study to better characterize the properties of intermediates micelles during the process of fibrils formation. Using light scattering techniques, Murphy and Pallitto (58) were able to monitor the temporal evolution of the average length of fibrils, the average number of monomers in a fibril, as well as to compute the time to appearance of macroscopic Aβ aggregates. Based on this characterization of the Aβ assemblies [e.g., monomers, micelles, filaments (i.e. thin fibrils), (thick) fibrils, and aggregates], Pallitto and Murphy (59) proposed a detailed multi-steps model for Aβ aggregation kinetics. In that model, a nucleation mechanism was not assumed for the initial step of conversion of unfolded monomers into micelles but for the further self-association of micelles into a nucleus (59). This multi-step mechanism also includes: (i) a cooperative (i.e., reversible) self-association of micelles into polymeric nucleus, (ii) elongation of nucleus into filaments by aggregation of micelles, (iii) lateral aggregation of filaments into fibrils, and (iv) fibril elongation *via* end-to-end aggregation (57, 59). As is usual in kinetic modeling, this multi-step mechanism translates into an infinite set of ODEs, which in turn, can be closed by summation to yield a set of eight ODEs. Note that the more types of Aβ assemblies included in the model, the greater the number of equations and kinetic constants rates. Fortunately, the master equations approach provides a unified framework for formulating kinetic equations for Aβ assemblies of any size (60, 61).

### Master Equations for Aggregation: A Modern Approach

The main idea underlying the master equations approach is to use principles of chemical kinetics in order to derive equations that explicitly account for the different microscopic processes involved in the proteins aggregation mechanisms (61). The ultimate goal is, from these (master) equations, to derive integrated rate laws that characterize the kinetics of protein aggregation. Although not explicitly developed in the specific context of Aβ, the master equations approach is currently established as the most general mathematical formulation for describing the kinetics of MP formation (60, 61).

Mathematical modeling of protein aggregation by master equations appeared in pioneering studies of filamentous growth phenomena (62). Under the basic principles of homogeneous nucleation, growth, and dissociation processes, Oosawa and Kasai (62) formulated a master equation for describing the time evolution of a population of filaments with different polymerization numbers. Similar to the NPM, the master equation is expressed by an infinite set of ODEs. It also relates directly to experimental measurements through the number and mass concentrations of the aggregates, which temporally evolve by a closed system of two ODEs, the so-called moment equations. Note that, in contrast to Masel et al. (26), the moment equations do not evolve as an epidemiological-like system. Instead, this system explicitly remarks its dependency on the concentration of free monomers. This discrepancy is given by the fact that, while Masel et al. (26) considered a free production of monomers at a constant rate, the master equations approach imposes the principle of conservation of mass. Thus, in the master equation approach, the time evolution corresponding to the monomer concentration only accounts for monomers consumed by incorporation into aggregates and monomers dissociated from aggregates, while keeping the total amount of monomers at a constant level (i.e., by conservation of mass).

Solving the moment equations yields the desired integrated rate laws that govern the reaction time course [see details in Ref. (61)]. Interestingly, a detailed analysis of the integrated rate law in Ref. (62) revealed that the actual role of the nucleation step was to generate new elongations seeds rather than facilitate the incorporation of monomers into aggregates. Even more, nucleation and growth processes seem to occur simultaneously [see a more detailed discussion in Ref. (61)], thus equally contributing to the length of the well-known lag phase observed on nucleation-dependent processes. In addition, for the very particular case of all proteins in initial monomeric configuration, Oosawa and Kasai (62) showed that the early stages of the reaction time course follow a quadratic rate law, which is a feature of growth governed by a primary nucleation pathway. The integrated rate law also revealed the dependency of the reaction time course on a single parameter (in terms of the microscopic rate constants), which ultimately scales with many of the phenomenological observable measurements (e.g. half polymerization time, maximal growth rate) (61). In other words, the integrated rate law of protein aggregation kinetics shows a scaling behavior. This feature has become a very important tool for understanding the protein aggregation process across different temporal and spatial scales [see an excellent discussion about this property in Ref. (63)].

During the last few decades, the Oosawa theory has been extended (60, 64–67) to include mechanisms of fragmentation and heterogeneous (secondary) nucleation (collectively called secondary pathways), where new aggregates could be also created at a rate depending on the concentration of existing aggregates. Extension of the Oosawa’s original formulation was mainly motivated by a discovery showing that the mass concentration of actin (65) and hemoglobin S (67) polymers tends to increase more rapidly than a quadratic rate law. As a consequence, the master equation should include extra terms for describing fragmentation processes and a secondary nucleation at a rate proportional to the surface area of existing aggregates (61, 64) (see Eq. A6 in Appendix in Supplementary Material). Similar to Oosawa, Ferrone et al. (66) derived closed equations for the time evolution of the number and mass concentration of the aggregates (see Eq. A7 in Appendix in Supplementary Material). Unfortunately, those equations are not analytically integrable, which poses additional difficulties for the derivation of the corresponding integrated rate law. As an alternative to the classical global analysis of the integrated rate law, Ferrone et al. (66) carried out a perturbation analysis for characterizing the early stages of the reaction time course [see a detailed explanation in Ref. (67)]. Such perturbation analysis revealed that the quadratic early-time growth rate predicted by Oosawa’s original theory is still valid when the reaction is dominated by the primary nucleation process. By contrast, when the reaction is dominated by the secondary nucleation process, the early-time growth follows an exponential law.

At that point, the applicability of an integrated rate law including fragmentation and secondary nucleation seemed to be limited to the early stages of the reaction time course. Evidently, an alternative solution would be employing numerical integrators for solving the closed system of equations corresponding to the number and mass concentration of the aggregates. However, it is not recommendable due to the highly non-linear structure of this system. Besides, understanding the role played by the kinetic rates is extremely difficult when only numerical solutions are available. In order to overcome these limitations, Knowles et al. (60) introduced a new technique that has been one of the major contributions to the kinetic theory of amyloid formation. Specifically, Knowles et al. (60) derived analytical solutions for the integrated rate law that extends its validity to the entire reaction time course. The main idea underlying this new technique is to use the early-times solution as a starting point in order to solve the non-linear moment equations with an iterative strategy [see details in Ref. (60)]. Thus, closed-form integrated rate laws were presented for the case of fragmentation (60) and monomer-dependent secondary nucleation (64). Similar to the Oosawa theory, these integrated rate laws revealed the dependency of the reaction time course on two parameters that can be easily related to experimentally observed phenomenological variables (60, 61, 64).

Having an analytical formula for the integrated rate law becomes extremely important since it allows global fitting [see an excellent discussion in Ref. (68)] of experimental data under different conditions (e.g., changing monomer concentrations). For instance, *in vitro* data corresponding to the peptides Aβ40 and Aβ42 fit very well to the theoretical model of Knowles et al. (60) which has clearly improved our understanding about the formation of Aβ aggregates (69–71). In fact, the analysis of such experimental data points to the secondary nucleation process as the mechanism responsible for the toxicity related to Aβ42 aggregation (69). Moreover, a similar analysis (70) showed clear differences (in the relative importance of primary nucleation versus the secondary nucleation) between the molecular mechanism of aggregation of Aβ40 and Aβ42.

During the last few years, the master equations approach and the corresponding integrated rate laws have been subject of multiple investigations (72–78). Among them, it is worth remarking the contribution of Cohen et al. (76) which included mechanisms of spatial propagation within the master equations framework. By fitting the model to experimental data, Cohen et al. (76) found that the secondary pathways govern the speed of spatial propagation by diffusion.

To conclude this section, note that, solely from the mathematical point of view, modeling the kinetics of protein aggregation through master equations is still a very active research field (79). Not to mention the profound impact that this novel approach has produced in the quest of therapeutic techniques for reducing the toxicity associated to low molecular weight Aβ aggregates (79).

### Coagulation Theory for Aggregation: A Road to Brain Imaging Modeling

As in the case of the master equations approach, the coagulation theory described by Smoluchowski’s equations (80) also covers the general case of self-association among particles assemblies of different sizes. The first references to Smoluchowski’s equations in the context of Aβ aggregation appeared in Ref. (58, 59) for describing the axial elongation of fibrils by end-to-end aggregation of shorter fibrils. By using Smoluchowski’s equations, Craft et al. (81) proposed a polymerization model where the nucleation process appears implicitly incorporated within the mechanism of association of small size polymers (e.g., monomers, micelles, and filaments). This model includes processes of production and dissociation of monomers as well as elongation and fragmentation processes for Aβ polymers. Similar to Ref. (34) for the case of prion diseases, Craft et al. (81) defined a polymerization ratio *R_0_* as the product of the production and elongation rates divided by the product of the degradation and fragmentation rates. The Aβ burden (i.e., total number of Aβ molecules) falls into a steady-state level if the polymerization ratio *R_0_* is less than one, and shows an increasing Aβ accumulation if this ratio is greater than one (81). A more formal presentation of the coagulation theory and Smoluchowski’s equations in the context of Aβ aggregation *in vitro* can be found in Ref. (82). (see Eq. A8 in Appendix in Supplementary Material).

An important turning point in the field of Aβ aggregation/propagation mechanism modeling is due to Achdou et al. (83). That study settled the grounds for linking molecular mechanism of early aggregation/propagation of Aβ oligomers with modern imaging techniques for measurements of amyloid deposition *in vivo*. The mathematical approach followed by Achdou et al. (83) was also based on Smoluchowski’s equations. However, rather than writing down closed ODEs for the moments of polymer length distribution, Achdou et al. (83) truncated the infinite set of differential equations (see Eq. A9 in Appendix in Supplementary Material) at a large enough number *N*. Under this approximation, large aggregates composed of more than *N* monomers are not supposed to coagulate each other. Thus, the time evolution equation corresponding to the truncation number *N* should be able to describe the summary of all Aβ assemblies composed of more than *N* monomers. In addition, Achdou et al. (83) realized that the Smoluchowski’s equations also provide a straightforward framework for incorporating processes of spatial propagation. Note, however, that Achdou’s model is only valid on small spatial domains (e.g., domain size comparable to a multiple of the size of a neuron), for which isotropic diffusion is a valid assumption. Under essentially the same assumptions, a straightforward generalization of Achdou’s model was given by Franchi and Tesi (84) which added fragmentation terms to equation (A9). As pointed out in Ref. (83) a major limitation is that this model is only able to simulate temporal trajectories up to the scale of microscopic processes occurring at the single neuron level.

The development of modern imaging techniques demands alternative models and the possibility to probe them at greater macroscopic scales. With this aim, a large-scale model was recently proposed by Bertsch et al. (85). Specifically, Bertsch et al. (85) coupled a set of truncated Smoluchowski’s equations to a kinetic-type transport equation that models the spreading of neuronal damage by neuron-to-neuron prion-like transmission. A major advantage of such modeling is the ability to incorporate two different temporal scales evolving over the same spatial domain: a rapid temporal scale (e.g. hours) for modeling microscopic processes of Aβ polymer agglomeration (by Smoluchowski’s equations); and a slow (e.g., months, years) scale to account for the longitudinal progression of AD (by the transport equation) (85). In that model, Bertsch et al. (85) assumed that oligomers of length greater than *N* can be measured by neuroimaging techniques (e.g., PIB-PET), as well as the parameter that controls the neuronal damage (e.g., FDG-PET). Note that Bertsch’s modeling approach is the first study that attempts to build a bridge between the microscopic and macroscopic processes that characterize the impact of Aβ aggregation on the onset and clinical progression of AD. Although still insufficient, some effort has been already put on checking the mathematical correctness and internal consistency of that model (86, 87).

### The Network Approach: Modeling Inter-regional Propagation

As it was already mentioned, modeling spatial spreading of prion proteins and MP by a homogeneous diffusion process is not a realistic choice in large spatial domains like the whole brain. Indeed, prion proteins and MP can spread long nerves and “infect” distant regions (44). Thus, for the very first time, Matthäus (43) used the so-called network approach for covering this scenario. There, a mathematical representation of a network consisted of a set of nodes and edges, where the nodes represented neuronal cells and the edges characterize whether two cells are connected (e.g., in the form of a synapse) or not (43). Using this simple mathematical framework, Matthäus (43) described the spread of prion protein infection along a network of inter-connected neurons by a discrete Susceptible-Infected epidemiological model (30). In this kind of models, the network nodes are classified into susceptible and infected nodes, where the susceptible ones become infected if at least one of their connected neighbors is already infected. Thus, in contrast to a homogeneous diffusional spread, the network approach models a rapid infection spread within clusters of highly connected neurons, and propagation to other clusters *via* long-distance connections (43).

The network approach (43) does not only model the influence of the network topology on the speed of the MPs spread but it is also flexible enough to handle different spatial scales. Correspondingly, Matthäus (88) formulated a system of reaction–diffusion equations by coupling kinetic equations of the heterodimer model with discrete diffusion terms to account for transport on networks. There, the network nodes can represent distant regions covering large spatial domains, thereby overcoming the limitations of Ref. (43). In this approach, the diffusion term at each node is spatially approximated by a sum of flows among neighboring nodes, such that the prion protein concentration is transported to the neighbors of the node and *vice versa* (88). Unfortunately, the reaction–diffusion equations formulated by Matthäus (88) have not been extended to nucleated polymerization mechanisms of prion replication.

While Matthäus (43, 88) modeled microscopic processes at the neuronal level, more recent macroscopic approaches have focused on the large-scale connectivity of the whole brain. In this line, Raj et al. (89) proposed a macroscopic network diffusion model (NDM). In the NDM, the number of MP afferents from a given brain region to any other region uniquely depends upon the disease concentration factors in both regions and upon the anatomical connection strength between them. This model was initially explored with structural atrophy data (89) and posteriorly with FDG PET metabolism (90), reproducing in both cases characteristic spatial distributions of MP effects on a relatively small sample of late-onset AD subjects. In addition, this diffusion model has been recently extended to account for impulsive sources of brain atrophy patterns over the brain connectivity network (91). As a main limitation, the NDM does not consider mechanisms of clearance and production of MP. Instead, the disease-related factors have no causal interpretation and accumulate gradually in the absence of any source and/or system resistance. By considering a more realistic scenario, Iturria-Medina et al. (92) introduced an epidemic spreading model (ESM) that simultaneously accounts for the regional capacity to produce/clean MPs and the topological information of the brain’s anatomical network (see Eq. 10). When applied to the study of late-onset AD using Aβ PET data, that model was able to reproduce Aβ deposition patterns at the individual level. In line with recent experimental results (93, 94), the ESM also identifies that reduced Aβ clearance, and not Aβ overproduction as the primary cause of Aβ deposition. Importantly, as highlighted in the ESM study (92), the cognitive and clinical states of the AD patients can only be partially explained by the mechanisms of Aβ production, clearance, and spatial propagation.

### Beyond MPs: An Integrative Modeling Approach

The existence of detailed pathological mechanisms and hypotheses for AD progression (49, 95–98) has motivated the consideration of a more integrative multifactorial modeling approach for MP formation and propagation (98–102). Early remarkable papers published by Edelstein-Keshet and Spiros (98) and Luca et al. (102) looked into detail at the underlying mechanism of deposition, uptake, removal, and degradation of Aβ. In particular, Edelstein-Keshet and Spiros (98) focused on modeling the interaction among Aβ deposits, glial cell, inflammation, and secreted cytokines, as well as the corresponding stress, recovery, and death of neuronal tissue. The numerical simulations carried out in Ref. (98) have helped to fill the gaps between hypothesized interactions and downstream consequences among different processes occurring during the AD progression. For instance, it was shown that an amyloid clearance deficiency could saturate the system and push it to toxic Aβ levels, yielding a state of competition between protective and toxic effects. Importantly, Edelstein-Keshet and Spiros (98) showed, for the very first time, a mathematical model where severity of AD does not need to correlate with sensitivity of neurons to amyloid fibers. In this way, Edelstein-Keshet and Spiros (98) highlighted the necessity of more integrative mathematical formulations that be able to consider AD and other neurodegenerative diseases as multifactorial, inter-dependent processes.

**Figure 3:**
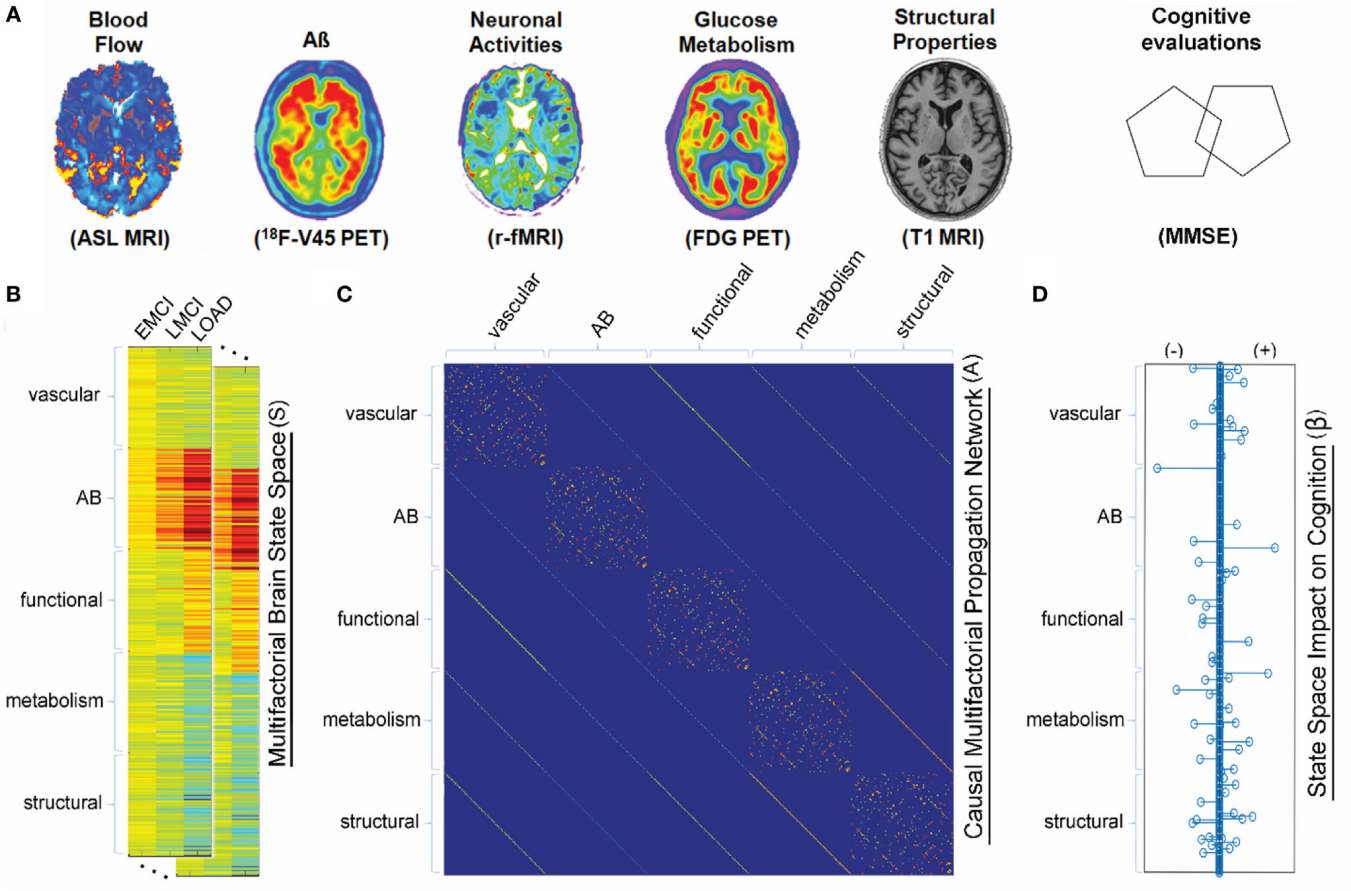
Multifactorial causal model. **(A)** Brain multimodal images and cognitive evaluations. **(B)** State space vectors (S), characterizing the brain’s multifactorial alteration levels with regard to a baseline. **(C)** Causal multifactorial propagation network capturing the direct interactions among regions (for each biological factor/imaging modality) or among factors (for each brain region). Diagonal blocks in the matrix correspond to a unique biological factor, with diagonal elements accounting for intra-regional effects and off-diagonal elements accounting for inter-regional alterations spreading across physical connections. Off-diagonal blocks correspond to the direct interactions between two different factors (e.g., glucose metabolism impact on tissue properties, or *vice versa*). **(D)** System has an output vector (β), representing the influence of the brain’s multifactorial state space on the cognitive state. Figure adapted from Iturria-Medina et al. (100) with permission of the journal.

In the same line, Puri and Li (99) presented a (microscopic scale) mathematical approach for describing the dynamics of critical components in AD pathogenesis. Formulated in terms of ODEs, that model describes well-known interactions among microglia, astroglia, neurons, and Aβ. The main feature there is to use neuronal death as a surrogate for senile amyloid plaque formation. Numerical simulations using that model indicates that inflammation might be used as an early biomarker for AD since microglia-related alterations can occurs long before apparent senile plaques formation (99). Similarly, Hao and Friedman (101) recently formulated a set of ODEs for describing microscopic processes of AD that included neurons, astrocytes, microglias, peripheral macrophages, as well as Aβ and hyperphosphorylated tau proteins. Based on numerical simulations, Hao and Friedman (101) suggested that a combination of inflammatory processes by cytokines and accumulation of Aβ plaques are key elements in accelerating the progression of AD. By following an integrative macroscopic approach, Iturria-Medina et al. (100) proposed a multifactorial causal model (MCM) in order to simultaneously account for macroscopic MP effects, regional multifactorial causal interactions, and pathological propagations through physical networks (e.g., axonal and vascular connectomes). The MCM (100) considers that, once a factor-specific event (e.g., Aβ deposition, vascular dysregulation, and structural alterations) occurs in a given brain region or in a set of regions, it can directly interact at the macroscopic level with other biological factors and alter their states. These alterations can also propagate through anatomical and vascular connections to other brain areas, where similar factor-factor and spreading mechanisms may occur in a positive feedback mechanism (see Figure 3 and Eq. 11).

## Therapeutic Intervention Modeling

Usually, chemical kinetic models of proteins aggregation are used as a surrogate to therapeutics interventions *in vitro*. The general idea is to simulate how a therapeutic intervention (e.g., drugs, antibodies, and molecular chaperones) might inhibit certain microscopic aggregation processes. Then kinetic rates of protein aggregation can be estimated and compared under both natural and inhibition conditions. For instance, monitoring kinetic rates as function of a hypothetical drug dose might help to extrapolate small drug dosages inherent of *in vitro* environments to dosages more closely resembling *in vivo* conditions (103).

**Figure 4:**
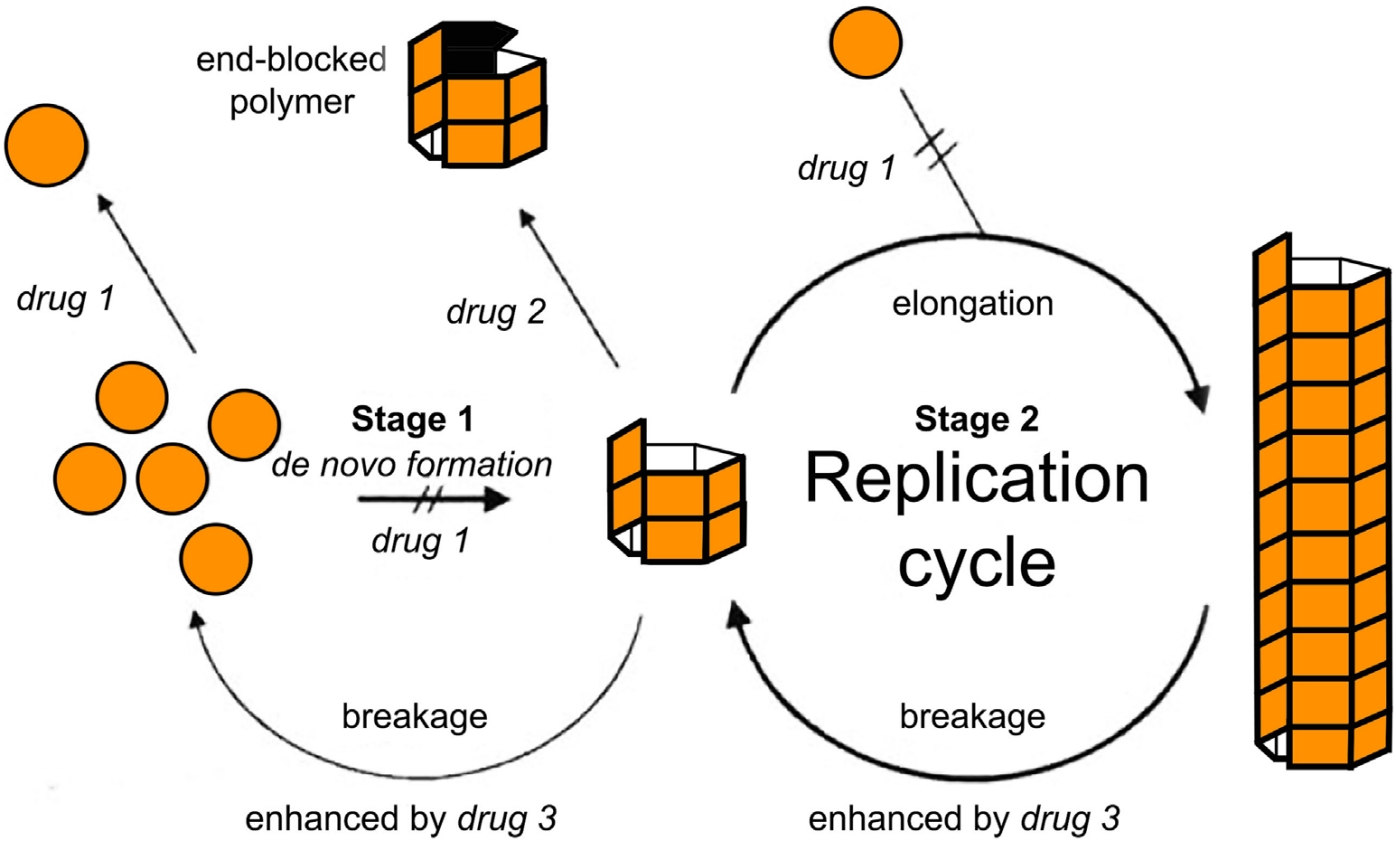
Tentative inhibition of amyloid aggregation/propagation by three different strategies (103). Drug 1 should lower the effective monomer concentration. Lowering the monomer concentration would inhibit *de novo* formation and slow down polymer elongation. Drug 2 should block growing polymer ends. In the context of macroscopically linear polymers, this would be equivalent to either blocking or capping the ends of the polymers. Drug 3 should increase the polymer breakage rate. Based on numerical simulations, Masel and Jansen (103) found that therapeutics following drug strategy (2) were the most promising ones, while the remaining strategies may be ineffective or even accelerate the amyloid formation process at low drug doses. Figure reproduced from Masel and Jansen (103) with permission of the journal.

Within the formalism of the simplest NPM (e.g., Eq. A3), two main strategies have simulated a therapeutic intervention on the kinetics of MPs (103). In this pioneering study, Masel and Jansen (103) used mathematical models to simulate the inhibition of amyloid propagation with three main approaches: (i) by lowering the effective monomer concentration of unfolded proteins; (ii) by blocking growing polymer ends; and (iii) by increasing the polymer breakage rate (see Figure 4). They found that therapeutics following the second strategy would provide promising results, while the remaining ones may be ineffective or even accelerate the amyloid formation process at low drug dosages (103). Indeed, any attempt of breaking up protein polymers into smaller pieces might yield undesired effects since small oligomers are more prone to propagate and spread neurotoxicity.

As discussed in a previous section, Craft et al. (81) established a non-linear relationship between the polymerization ratio *R_0_* and the total Aβ burden, where *R_0_* determines two different regimes: a steady-state of Aβ burden or a super-critical regime of continuous Aβ burden increase. By using an empirical relationship between Aβ burden and clinical dementia scores (CDR), Craft et al. (81) explored a potential therapeutic treatment based on the reduction of the polymerization ratio. Since the polymerization ratio depends on four different kinetic rates, several treatment strategies could be easily simulated in this context: (i) the reduction of Aβ monomers production rate, (ii) the enhancement of fragmentation, (iii) the enhancement of the clearance (i.e., degradation) rate, and (iv) the reduction of the elongation rate. In fact, around the time that paper was originally submitted [although originally submitted in 2001 (81), only appeared published in 2005], several treatment approaches following some of these strategies appeared promising in preclinical studies (104–106).

Note that the assessment of different treatments scenarios is usually carried out in two main steps. First, one assumes that a hypothetical change (e.g., by treatment) on the appropriate kinetic rates and then substitutes those modified rates into the original model in order to evaluate the post-treatment dynamical states. The therapeutic treatments simulations carried out in Ref. (81) suggested three different possible outcomes: (1) a reduction of Aβ burden from a pre-treatment steady state to a post-treatment steady state; (2) a transition from a pre-treatment Aβ burden increasing state to a post-treatment steady state; and (3) a reduction in Aβ burden increasing from a pre-treatment super-critical state to a post-treatment super-critical state. Importantly, as pointed out by Craft et al. (81), the failure of a potential drug to reduce the total Aβ burden may not necessarily be associated to drug inactivity but rather to a late intervention during the super-critical state. Besides, Craft et al. (81) showed that any drug treatment based on clearance rate enhancers might be more effective in reducing the total Aβ burden than those based on polymer fragmentation enhancers.

The previous therapeutic intervention modeling was generalized in Ref. (107) by the simulation of the accumulation and spreading of Aβ among the brain, CSF, and plasma. Note that the proposed compartmental model does not only consider production and degradation of Aβ polymers within the brain but also accounts for sources and losses due to transport to and from the plasma and CSF (107). The numerical simulations carried out in Ref. (107) suggest that potential drugs based on the production of Aβ inhibitors (e.g., by enhancing clearance rate) are likely to reduce Aβ burden in the brain, CSF and plasma. By contrast, any drug based on favoring polymers fragmentation and blocking polymers elongation may also reduce Aβ burden in the brain but may not reduce (or even cause a subtle transient increase) Aβ levels in CSF and plasma. By following similar ideas, Das et al. (108) proposed a two-compartment model for the distribution of the γ-secretase inhibitor between the plasma and the CSF, and its effect on the Aβ concentrations in the two compartments. The steady-state analysis of this model reproduced a primary γ-secretase inhibitor effect that caused a decrease in Aβ concentration in both CSF and plasma. However, the model also captured an overshoot of Aβ in the plasma compartment, which was explained by an off-target effect (attenuation of the Aβ clearance rate) of the γ-secretase inhibitor. Das et al. (108) concluded that any effective Aβ-reducing drug would have to necessarily account for more detailed kinetic mechanisms of Aβ production and clearance.

More recent studies such as Ref. (109, 110) used a stochastic modeling approach for simulating discrete versions of simple ODEs describing MP aggregation processes. Similar to Ref. (81), Proctor et al. (109) showed that a small decrease in the dissociation rate of Aβ monomers is enough to increase the chance of appearance of intermediate Aβ toxic species (e.g., dimers, oligomers). In addition, numerical simulations showed that any potential antibodies therapy against the formation of Aβ dimers would have large benefits as an early intervention strategy. Similarly, Proctor et al. (110) studied a model that accounts for a simultaneous intervention on Aβ and tau pathology. Thus, Proctor et al. (110) was able to show that therapies based on Aβ immunization would not only be able to reduce the amount of senile plaques but also produce small reductions in levels of soluble Aβ species, phosphorylated tau proteins, and neurofibrillary tangles.

Undoubtedly, a renovated interest on therapeutic intervention modeling has been motivated by the recent advances in the field of chemical kinetics and protein aggregation (111–117). In Ref. (116), Arosio et al. used the chemical kinetic approach to model the interaction between molecular chaperones and different protein species. The main idea there was to identify which microscopic reaction steps (i.e., primary nucleation, elongation, fragmentation, and secondary nucleation) where perturbed by the binding of the molecular chaperones to certain protein species (116). By using the master equations approach and the corresponding reaction profile for the total fibril mass concentration, Arosio et al. (116) compared the estimated kinetic rate constants in the absence and presence of different concentrations of molecular chaperones. Note that this kinetics profile analysis required a new mathematical development [See the Supplementary in the external Ref. (116)] for extending the master/moments equations formalism for modeling the binding between molecular chaperones and different protein species (e.g., monomers, fibril end, and fibril surface). For the particular case of the Aβ42 protein, such analysis revealed that the action of particular molecular chaperone (termed DNAJB6) inhibits the primary nucleation process. By contrast, the presence of another molecular chaperone (termed proSP-C Brichos) produces a reduction in the secondary nucleation rate (116). Note that Arosio et al. (116) also analyzed more complex scenarios where specific molecular chaperones might simultaneously affect different microscopic processes (e.g., elongation and secondary nucleation) that characterize the aggregation of the Aβ42 protein. In summary, Arosio et al. (116) provided a detailed modeling of different combination of mechanisms through which molecular chaperones might suppress amyloid aggregation. These results open up a research avenue where molecular chaperones and other classes of compound might be used as potential therapeutic agents in MP-related diseases.

Similarly, Habchi et al. (113) used the chemical kinetics approach for developing a rational drug discovery strategy that takes into account the specific microscopic steps in the aggregation of the Aβ42 protein. Analogous to the case of molecular chaperones, a potential drug compound could bind to different species of Aβ42 and selectively affect specific microscopic steps during the aggregation process (113). The strategy then proceeds by monitoring the kinetic profiles of Aβ42 fibril formation in the absence and presence of particular potential drugs. Remarkably, by following the master equations modeling approach, Habchi et al. (113) reported that an anticancer drug (termed bexarotene) disturbs the primary nucleation step in the Aβ42 aggregation, delays the formation of toxic oligomers and completely suppresses Aβ42 deposition. This is a general framework that yields a systematic drug discovery strategy aimed to identify a variety of small molecules (112) and antibodies (115) that not only target the onset of aggregation but also the secondary nucleation step responsible for the proliferation of toxic Aβ42 oligomers.

Unfortunately, large-scale models based on phenomenological imaging-based features of protein aggregation (e.g., models based on the brain network approach) have not yet taken advantage of the outstanding advances on therapeutic interventions using the chemical kinetics approach. In this direction, Iturria-Medina et al. (100) used a theoretical control analysis to predict multifactorial intervention effects required to revert brain biomarkers from an advanced disease stage to a clinical normal state. In particular, Iturria-Medina et al. (100) used a multifactorial causal model as an *in silico* evaluator for comparing the macroscopic effects of multiple possible interventional treatments. Their results provided an efficient ranking of multiple AD interventions, which might explain why recent single-target Aβ-based therapies failed to improve clinical outcomes in AD (118, 119).

## Conclusion

In this paper, we intended to provide an historical overview of the development of mathematical models for aggregation and propagation of MP in neurological diseases. Our main goal was to put in contact the neuroimaging community with important studies of MP chemical kinetics modeling, which have not been traditionally acknowledged but constitute a solid framework for a better understanding of neurological diseases evolution. We have mostly followed a chronological presentation of only those mathematical models that, in our opinion, established turning points from either the mathematical modeling point of view or the ability to describe truly biological processes. As a summary of our presentation, we selected the most important of those contributions and present them in Table 1. Note that in the main text, we have barely presented an overview of the mathematical formulation and the corresponding biological interpretation involved in those models. Besides, in order to facilitate our exposition, we have used a unified mathematical notation that, in some cases, might differ from the original formulation (see Appendix in Supplementary Material).

**Table 1:** Summary of the most significant studies presented in the manuscript. These studies established turning points from either, the mathematical modeling point of view or the ability to describe truly biological processes.

Study	Main features	Relevance	Validation
Oosawa and Kasai (62)	– Infinite set of ODEs – The system can be closed to a moment equations model – Analytical expressions for integrated rate laws based on a primary nucleation mechanism	– Introduced the master equations formalism for protein aggregation modeling	– Validation with actual *in vitro* data of different proteins
Nowak et al. (28)	– Infinite set of ODEs – The system can be closed to an epidemiological-like model – Steady states described by a reproductive ratio constant – Does not consider spatial spreading	– The first reference to analogy with epidemiological-like systems	– Numerical simulations of prion diseases
Masel et al. (26)	– Infinite set of ODEs – Nucleated polymerization model considers the formation of a nucleated seed of critical size – The system can be closed to an epidemiological-like model – Steady states described by a reproductive ratio constant – Does not consider spatial spreading	– Description of quantification of kinetics constants using actual data	– Validation with actual *in vitro* data of prion diseases
Masel and Jansen (103)	– Infinite set of ODEs – The system can be closed to an epidemiological-like model – Does consider the inhibition of amyloid propagation – Does not consider spatial spreading	– The first approach to therapeutic intervention from the modeling point of view	– Numerical simulations of drugs effects on prion and amyloid-related diseases
Stumpf and Krakauer (45)	– Epidemiological-like system of ODEs – Does consider spatial spreading	– The first time attempt to account for effects of local neuronal connectivity	– Numerical simulations of prion diseases
Craft et al., (81)	– Infinite set of ODEs – No explicit specification of an intermediate nucleation mechanism – Steady states described by a reproductive ratio constant	– The first attempt of describing drugs through a steady-state analysis – Showed for the very first time the potential effectiveness of drug treatments based on clearance rate enhancers	– Numerical simulations of potential therapeutic treatments for reduction of Aβ burden
Greer et al. (33)	– Finite set of PDEs – The system can be closed to an epidemiological-like model – Steady states described by a reproductive ratio constant – Does not consider spatial spreading	– The PDE formalism improved the mathematical analysis as compared to the infinite set of ODEs – A more detailed characterization of the epidemiological-like behavior of the NMP	– Validation with actual *in vitro* data of prion diseases
Matthäus (43)	– Infinite set of partial differential equations (PDEs) with diffusion terms – Does consider spatial spreading in small 1D domains – Does consider epidemiological-like models on macroscopic large-scale networks	– The first reference to macroscopic models using the network approach	– Validation with actual *in vitro* data of prion diseases – Simulation of prion spread in the mouse visual system
Knowles et al. (60)	– Infinite set of ODEs – The system can be closed to a moment equations model – Analytical expressions for integrated rate laws that account for mechanisms of fragmentation and secondary nucleation	– Detailed characterization of the protein aggregation kinetics by explicit expressions of integrated rate laws – The integrated rate laws are valid for the entire time course reaction	– Validation with actual *in vitro* data of different proteins
Achdou et al. (83)	– Finite set of PDEs with diffusion terms – Does consider spatial spreading in small 3D domains	– The first attempt of linking kinetics Aβ formation and propagation with modern imaging techniques for measurements of amyloid deposition *in vivo*	– Numerical simulations of Aβ in Alzheimer’s disease
Iturria-Medina et al. (92)	– Epidemiological-like system of ODEs – Does consider spatial spreading – Does consider the actual large-scale topology of brain networks – Does consider mechanism for regional production and clearance of misfolded protein (MP)	– The first computational model highlighting the direct link between structural brain networks, production/clearance of MP – The first model validation through parameter estimation from actual imaging data	– Validation through numerical estimation of model parameters from actual amyloid PET data
Bertsch et al. (85)	– Finite set of PDEs with diffusion terms couple to a kinetic-type transport equation – -Does consider spatial spreading in small 3D domains	– The first attempt to simultaneously modeling microscopic and macroscopic effects of Aβ propagation – Incorporation of two different temporal scales evolving over the same spatial domain	– Numerical simulations of Aβ in Alzheimer’s disease – Empirical comparisons with actual PET data
Habchi et al. (112)	– Infinite set of ODEs – The system can be closed to a moment equations model – Analytical expressions for integrated rate laws that account for mechanisms of fragmentation and secondary nucleation	– Introduced a rational drug discovery strategy based on the master equations formalism – Discovery of small molecules that inhibit specific microscopic steps of Aβ42 aggregation	– Validation with actual *in vitro* Aβ42 data

We mainly focused our presentation on those models that simulate microscopic processes of nucleation-dependent mechanisms of MP formation. These kinds of (microscopic scale) models provide a unique theoretical framework for relating microscopic processes to macroscopic kinetic profiles of protein aggregation. Thus, the most accepted model for the formation of protein aggregates relies on a variety of microscopic processes, including primary nucleation, fibril elongation, fibril fragmentation, and secondary nucleation, which are collectively summarized by a macroscopic kinetic profile that follows a characteristic sigmoidal shape. The procedure by which highly reproducible kinetic measurements are fitted to this sigmoidal profile allows for (i) detailed characterization of protein aggregation mechanisms in terms of underlying molecular events and (ii) the development of drugs and early therapeutic interventions that might inhibit some of those molecular events. Among other lessons, we have learned that an increase in the monomer concentration of MPs as well a reduction of the monomers clearance rate yield an increase in the growth rate of amyloid formation. This simple lesson highlighted the importance of systematically incorporating chemical kinetics models into strategies of drugs discovery. In fact, it has been suggested (112) that the failure of current therapeutic strategies against AD can be attributed to a limited understanding of the molecular mechanisms by which the tested compounds interact with different species of protein aggregates.

On the other hand, we also presented macroscopic scale modeling approaches that mainly account for the large-scale connectivity of the brain and the indirect phenomenological mapping of the underlying molecular mechanisms of protein aggregation. In line with the network degeneration hypothesis (25), the macroscopic NDM of Ref. (89) supported that MP propagation follows disease-specific anatomical patterns. Similarly, the ESM of Ref. (92) highlighted the importance of considering intra-regional MP generation/clearance and the inter-regional spreading through the anatomical connections. In addition, by using a multifactorial causal model, Iturria-Medina et al. (100) concluded that late-onset AD it is not caused by a unique dominant biological factor (e.g., vascular or Aβ deposition) but by the complex interplay among multiple relevant biological interactions. Taken together, those large-scale mathematical models point to a lack of an integrative perspective as the main cause for the failure of therapeutic strategies against AD.

Undoubtedly, there is still a gap to fill for properly modeling underlying microscopic processes of protein aggregation and their effects on the progression of the associated neurological diseases, as measured by *in vivo* imaging techniques and the assessment of the patient’s cognitive condition. Indeed, despite recent efforts (85), most of the large-scale models for protein aggregation still need to incorporate additional components in order to deal with two different temporal scales and spatial domains. Namely, the small-scale where microscopic aggregation processes occur relatively fast and the large-scale where protein deposits accumulate over a long-time period. We hope that future studies about pharmacokinetic modeling of *in vivo* protein binding using PET imaging might shed light on those unresolved issues and yield systematic drug discovery strategies under a broader, integrative perspective.

## Author Contributions

FC and YI-M conceived the review and wrote the manuscript. YI-M prepared the figures. AE revised the manuscript. All authors contributed to constructive discussions during the manuscript preparation.

## Conflict of Interest Statement

The authors declare that the research was conducted in the absence of any commercial or financial relationships that could be construed as a potential conflict of interest.
