# Supplementary material for: Mathematical Modeling of Protein Misfolding Mechanisms in Neurological Diseases: A Historical Overview
Source: Front Neurol. 2018 Feb 2;9:37. doi: 10.3389/fneur.2018.00037 (PMC5801313; doi:10.3389/fneur.2018.00037)
Supplement: Supplementary file 1 [file data_sheet_1.PDF]

Appendix

Nucleated Polymerization Model

Let u and v_i denote PrP^C monomers and aggregates of PrP^{Sc} composed of i monomers, respectively. The classical nucleated polymerization model is described by the following system of ODEs (see Appendix A in (27)):

$$\dot{u} = \lambda - du - \beta \sum_{i=1}^{\infty} uv_i \qquad \text{(A1)}$$

$$\dot{v_i} = \beta u(v_{i-1} - v_i) - av_i - b(i-1)v_i + 2b \sum_{j=i+1}^{\infty} v_j\,, \; i = 2,3,\ldots,$$

where the kinetic constants *λ, d, β, b, a* denote the rates of PrP^C monomers production (by genetic machinery), metabolic degradation of monomers PrP^C, aggregation of PrP^C monomers to PrP^{Sc} oligomer (i.e. polymerization), fragmentation of PrP^{Sc} aggregates and metabolic degradation of PrP^{Sc} aggregates, respectively.

By summing over all i, yields an ODEs system of only three equations (27):

$$\dot{x} = \lambda - dx - \beta xy \qquad \text{(A2)}$$

$$\dot{y} = bz - (a+b)y$$

$$\dot{z} = \beta xy - az,$$

where $x=u$, $y = \sum_{i=1}^{\infty} v_i$ and $z = \sum_{i=1}^{\infty} iv_i$ denote the abundance of PrP^C protein, the total length of PrP^{Sc} aggregates and the total mass (i.e. first moment of the length distribution) of PrP^{Sc} protein, respectively.

Nucleated Polymerization Model with critical threshold size

The extended NPM can be described by the system of ODEs (25):

$$\dot{x} = \lambda - dx - \beta xy + n_0(n_0 - 1)by \qquad \text{(A3)}$$

$$\dot{y} = -ay + bz - (2n_0 - 1)by$$

$$\dot{z} = \beta xy - az - n_0(n_0 - 1)by,$$

where n_0 denotes the critical threshold size.

Nucleated Polymerization model with spatial diffusion

The incorporation of a diffusion component to the discrete NPM is given by the following infinite set of PDEs (43):

$$\frac{\partial u}{\partial t} = \lambda - du - \beta \sum_{i=1}^{\infty} uv_i + 2b \sum_{i=1}^{n_0-1} \sum_{j=i+1}^{\infty} iv_j + D_u \frac{\partial^2 u}{\partial s^2} \qquad \text{(A4)}$$

$$\frac{\partial v_i}{\partial t} = \beta x(v_{i-1} - v_i) - av_i - b(i-1)v_i + 2b \sum_{j=i+1}^{\infty} v_j + D_i \frac{\partial^2 v_i}{\partial s^2}, \; i = 2,3,\ldots,$$

where the variables $u=u(t,s)$ and $v_i=v_i(t,s)$ also depend on the spatial component s, defined over a one-dimensional bounded domain, and D_u, D_i denote diffusion coefficients.

Nucleated Polymerization model with connectivity features

Incorporation of connectivity features into an epidemiological-like model is given by (45):

$$\dot{x}_i = \lambda_i - d_i x - \beta x_i y_i, \qquad \text{(A5)}$$

$$\dot{y}_i = \beta x_i y_i - \sum_{i=1}^{N} (c_{ij} y_i - c_{ji} y_j) - \gamma y_i,$$

$$\dot{z}_i = \gamma y_i,$$

where x, y and z denote concentrations of PrP^C, PrP^{Sc} and extracellular amyloid plaques, and the index $i=1,\ldots,N$ labels the ensemble of N connected cells. Here the constant γ represents the rate of PrP^{Sc} into amyloid plaques, and the connectivity strengths c_{ij} are defined as a function of both, the Euclidean distance between the cells i and j and the connectivity structure of the ensemble of cells. The constants λ_i, d_i and β_i denote the kinetic rates of PrP^C production, PrP^C degradation, and conversion of PrP^C into PrP^{Sc}, respectively.

Master equations for protein aggregation

The master equation for the time evolution of protein concentrations of monomers and length-i species is given by the system of ODEs (64):

$$\dot{u} = -\frac{d}{dt}\sum_{j=n_c}^{\infty} jv_j \text{ (A6)}$$

$$\dot{v_i} = 2k_+u(v_{i-1} - v_i) + 2k_{off}\,u(v_{i+1} - v_i) - k_-(i-1)v_i + 2k_-\sum_{j=i+1}^{\infty} v_j +$$

$$\delta_{i,n_c}k_n u^{n_c} + \delta_{i,n_2}k_2 u^{n_2}\sum_{j=n_c}^{\infty} jv_j\ ,\ i = 2,3,\ldots,$$

where k_+, k_{off}, k_-, k_n and k_2 denote the kinetic rates for elongation, dissociation, fragmentation, primary nucleation and secondary nucleation, respectively. There, n_c and n_2 denote the reaction orders for the primary and secondary nucleation, respectively, and δ represents the Kronecker function.

The kinetic (moment) equations for the number and mass concentrations are given by

$$\dot{P} = k_-(M - (2n_c - 1)P) + k_2u(t)^{n_2}M + k_nu(t)^{n_c} \quad \text{(A7)}$$

$$\dot{M} = 2(u(t)k_+ - 2k_{off} - k_-n_c(n_c - 1)/2)P + n_2k_2u(t)^{n_2}M + n_ck_nu(t)^{n_c}$$

Smoluchowski's equations for polymers aggregation

The Smoluchowski's equations for polymers aggregation is given by (82):

$$\dot{v_i} = \frac{1}{2}\sum_{j+k=i} c_{j,k}v_jv_k - v_i\sum_j c_{i,j}v_j\ ,\ i = 1,2,\ldots, \quad \text{(A8)}$$

where $c_{i,j}$ denotes the kinetic rate of association between two polymers of sizes i and j.

Truncated system of Smoluchowski's equations with diffusion

The truncated system of Smoluchowski's equations can be written as a discrete system of PDEs (83):

$$\frac{\partial v_i}{\partial t} = D_i\nabla^2 v_i + \sum_{j=1}^{i-1} c_{j,i-j}v_jv_{i-j} - v_i\sum_{j=1}^{N} c_{i,j}v_j\ ,\ i = 1,2,\ldots,N \quad \text{(A9)}$$

where $v_i=v_i(t,s)$ depend on a (multi-dimensional) spatial component s and D_i denote diffusion coefficients.

Epidemic Spreading Model (ESM) of MP generation, degradation and propagation

The temporal evolution of the MP burden probability is described by the system of ODEs (92):

$$\frac{dP_i}{dt} = (1 - P_i)\left(\sum_{j\neq i} c_{j,i}\beta_j^{ext}(t)P_j + c_{i,i}\beta_i^{int}(t)P_i\right) - e^{-\delta P_i}P_i + \aleph, \qquad \text{(A10)}$$

with

$$\beta_i^{ext}(t) = g(t)\left(1 - e^{-\beta P_i(t)}\right)$$

$$\beta_i^{int}(t) = (1 - g(t))(1 - e^{-\beta P_i(t)}),$$

where $c_{i,j}$ denotes the (asymmetrical) weighted anatomical connection probability between the regions j and i (e.g. obtained by diffusion MRI tractography techniques) and β, δ denote the MP production and clearing rate, respectively. Here, the function g denotes the Gini coefficient that accounts for the statistical dispersion of the system and ℵ is an additive Gaussian noise term.

Multifactorial Causal Model (MCM)

The dynamic behavior for the alteration/disequilibrium of several biological factors relative to the baseline state is described by the system of ODEs(100):

$$\dot{x}_i^m = \sum_{n=1}^{M} \alpha^{n.m} x_i^n + \sum_{\substack{j=1,\\ j\neq i}}^{N} c_{j,i}^m(t)\beta^m x_j^m - \sum_{\substack{j=1,\\ j\neq i}}^{N} c_{i,j}^m(t)\beta^m x_i^m, \qquad \text{(A11)}$$

where M and N denote the number or biological factors and the number of macroscopic brain regions, respectively. Here, the parameters β^m and $\alpha^{n,m}$ control the propagation weight for each factor m and the direct intra-regional impact of the factor n over factor m, respectively. The influence of network connectivity on each factor m has been defined through the parametric functions $c_{i,j}^m(t)$, where the factor's alteration is assumed to propagate across anatomical and/or vascular networks.
